# Sequence co-evolutionary information is a natural partner to minimally-frustrated models of biomolecular dynamics

**DOI:** 10.12688/f1000research.7186.1

**Published:** 2016-01-26

**Authors:** Jeffrey K Noel, Faruck Morcos, Jose N Onuchic

**Affiliations:** 1 Center for Theoretical Biological Physics, Rice University, Houston, TX, USA; 2 Kristallographie, Max-Delbrück-Centrum für Molekulare Medizin, Berlin, Germany; 3 Department of Biological Sciences, University of Texas at Dallas, Richardson, TX, USA

**Keywords:** minimally frustrated models, biomolecular dynamics, frustrated protein models, protein structure model, x-ray crystallography, nuclear magnetic resonance, Direct coupling analysis

## Abstract

Experimentally derived structural constraints have been crucial to the implementation of computational models of biomolecular dynamics. For example, not only does crystallography provide essential starting points for molecular simulations but also high-resolution structures permit for parameterization of simplified models. Since the energy landscapes for proteins and other biomolecules have been shown to be minimally frustrated and therefore funneled, these structure-based models have played a major role in understanding the mechanisms governing folding and many functions of these systems. Structural information, however, may be limited in many interesting cases. Recently, the statistical analysis of residue co-evolution in families of protein sequences has provided a complementary method of discovering residue-residue contact interactions involved in functional configurations. These functional configurations are often transient and difficult to capture experimentally. Thus, co-evolutionary information can be merged with that available for experimentally characterized low free-energy structures, in order to more fully capture the true underlying biomolecular energy landscape.

## Introduction

High-resolution structural techniques (e.g., X-ray crystallography and nuclear magnetic resonance) have provided the data necessary to develop and refine a multitude of potential energy functions used in the simulation of biomolecules. In particular, these structures provide the parameterization for simplified models that are based on the energy landscape theory of protein folding. These models construct an energetically unfrustrated (ideal) funneled landscape by including stabilizing interactions between native contacts (i.e., amino acid pairs that are nearby in the three-dimensional native structure of a protein). In cases in which experimental structures are lacking or insufficient, it becomes necessary to supplement these models with other sources of contact information. An emerging technique for contact estimation is via the statistical analysis of residue co-evolution in families of protein sequences. Combinations of high-resolution structural data and predictions from residue co-evolution are proving to be invaluable tools for building models to study protein structure and dynamics.

Understanding the fundamental process of how a heterogeneous polypeptide can reversibly fold into a distinct native three-dimensional structure on biological timescales led to the development of the energy landscape theory of biomolecular folding. This theory is based on the principle of minimal frustration ^1^ and the folding funnel concept ^2, 3^. These physical principles describe an energy landscape that has been molded by evolution such that the native interactions (i.e., the molecular interactions present in low free-energy configurations of folded proteins and RNAs) are, on average, more stabilizing than non-native interactions. The consequence of proteins having sufficiently reduced energetic frustration is that geometry dominates energetic roughness in determining folding mechanisms. Thus, a description of the effective energetics of the folding phenomenon can be attained by including a set of native stabilizing interactions consistent with the native basin of attraction. Potential energy functions of this type, which use experimental information to determine such native interactions, are known as “structure-based models” (SBMs) ^4– 6^ and, when employed in dynamical models, are powerful tools for understanding the connection between structure, folding, and function. Although these SBMs have been successfully applied to different biomolecules, we will be focusing on proteins for clarity in this review.

Structural information, however, may be limited for many interesting systems. This is particularly true for functional configurations that are transient or partially disordered or both. The recent explosion in genomic information has enabled complementary methods for discovering functionally important amino acid interactions. The minimal frustration principle applies equally to any sequence of amino acids that can robustly fold to a particular native structure. Thus, in a family of sequences where most of them fold to a common structure, residue positions that are in contact will display a correlated mutational record because of the global evolutionary constraint that the native structure imposes for foldability. Of course, additional constraints beyond folding affect sequence evolution, including maintenance of molecular assemblies, enzymatic activity, and allosteric motions. Signals of these functionally relevant contacts are necessarily mixed with those providing robust folding. To identify such relevant interactions involved in folding and function, a number of methodologies have been developed in recent years that have been successful in uncovering such molecular couplings from sequence data. One of them is direct coupling analysis (DCA) ^7, 8^, which is designed to infer a global statistical model from a multiple sequence alignment (MSA) of a single protein family. Using a maximum entropy approach, DCA infers the parameters of an effective energy function consisting of single-site fields and pairwise couplings that is able to approximately reproduce the empirically observed single-site and pairwise amino acid frequencies from the input sequence alignment. The DCA energy function is known as a Potts model, a generalized Ising model that includes non-nearest neighbor interactions and non-constant spin-spin interactions. In practice, couplings of varying strength are computed between all possible pairs of sequence positions. In the past, accurate and tractable approximations of such global models were elusive and detection of direct correlations, as opposed to an aggregate of direct and indirect correlations, was challenging. Other methods are derived from similar theoretical perspectives but have varying computational demands and accuracies ^9– 12^. Using an inferred effective energy function, one can estimate pairwise direct probabilities at a particular pair of residue sites. Calculating the Kullback–Leibler divergence between these joint probabilities and single marginal frequencies gives the direct information (DI) score for that residue pair. DI is a proxy of how “directly correlated” two sites are in an MSA. When compared with crystal structures, high DI scores correlate highly with native contacts, and more than 80% overlap, on average, for the top residue pairs in many protein families ^7, 13^. The full set of highly scoring contacts amounts to a superset of minimally frustrated and functionally important residue pairs that are spatially localized in the functional configurations of the members of a protein family. Here, we will review the current progress in using residue co-evolution for modeling the structure and dynamics of proteins with a focus on its combination with SBMs.

## Residue co-evolutionary constraints are natural input for minimally frustrated protein models

In their simplest form, SBMs idealize minimally frustrated protein energy landscapes by including only native interactions. This model removes any residual non-native energetic roughness and clarifies analysis of the geometrical and topological aspects of protein dynamics and folding. These models faithfully represent the local geometry through bond, angle, dihedral, and excluded volume terms at either single-bead-per-residue or all-atom resolutions. Non-local interactions consist of stabilizing pairwise potentials applied between residue (or atom) pairs that are nearby in the native structure. These pairwise interactions are called native contacts, and the entire set is known as a native contact map. All of the interactions, local and non-local, are set to have an explicit minimum at the native structure, hence the name “structure-based”. The simplified construction of the potential energy function permits for reduced computational requirements, and the explicitly encoded native interactions provide a baseline model that can be used for molecular modeling or studying physical perturbations. For a detailed discussion of the theoretical foundation and construction of SBMs, we refer you to the following reviews, ^14– 16^, and the references therein.

The quality of contact maps derived from DCA and similar methods have been benchmarked against contact maps calculated from crystallographic structures, and their accuracy is promising. In general, the larger and more diverse the family of sequences, the higher the quality of contact prediction. The high level of DCA accuracy provided sufficient tertiary constraints to allow folding single domain proteins to within 3 Å from the crystal structure when given knowledge of the secondary structure ^17– 21^. A rule of thumb is that the number of sequences should be larger than 1000 with less than 80% identity; however, others propose an even lower requirement of a minimum number of sequences close to the length L of the protein polypeptide chain, provided that they are diverse ^18^. The notoriously difficult problem of predicting membrane protein structures has also been aided by considering evolutionarily coupled pairs ^22, 23^.

A native contact map derived from a single native structure is often not sufficient to encode all the functionally relevant, minimally frustrated interactions. This led to the development of a variety of “multi-basin” models, where multiple experimental structures or structural constraints are included in a single SBM ^24– 26^. As described above, residue pairs with the highest DI scores, the high DI pairs (HDPs), are consistent with the native contact maps. Thus, in an analogous fashion, predicted contact constraints from co-evolution can be merged with contact maps computed from experimental structures in order to more fully capture the true underlying biomolecular energy landscape, including functional transitions and conformations, and therefore to be consistent with multiple structures ^27– 29^.

## Recent advances

Interactions between proteins are fundamental to cellular processes. Where these interactions involve direct contact, multimeric structures, both long-lived and transient, leave correlated mutational patterns between interacting surface residues. A pioneering study used the HDPs between a histidine kinase and its response regulator to make a prediction of the transient protein complex enabling phosphotransfer ^30^. This allowed a prediction for the histidine kinase TM0853 and its response regulator TM0468 that was later confirmed experimentally to be within 3.3 Å ^31^. These predictions are made by minimizing a contact-based energy function consisting of dimeric HDPs. Where dimerization only weakly perturbs the monomer structure, refined rigid-body modeling in combination with co-evolutionary constrains can be employed to estimate protein complexes. When combined with experimental observations, directly coupled amino acids can unveil protein interfaces relevant for the study of disease ^32^. Larger monomer distortions can be readily sampled with SBMs coupled with simulated annealing ^33^. Current protocols involving HDPs have allowed the large-scale prediction of both homodimers ^34^ and heterodimers ^35, 36^. The HDP contact map for a protein family that forms homodimers is a prime example of how ambiguity can arise in co-evolutionary information. The co-evolving dimeric interfacial contacts are mixed with HDPs selected for monomeric folding, but the dimeric contacts can in general be sorted from the monomeric contacts when there is a known monomer structure ^34^. But rarely are there true dichotomies in biology; the existence of domain swapping ^37, 38^ and structural symmetry ^26, 39^ highlights some difficulties in assigning particular roles to each HDP. Also, some protein-protein interactions are mediated by disordered regions that order upon binding. The utility of DCA in these cases remains to be tested.

**Figure 1.**
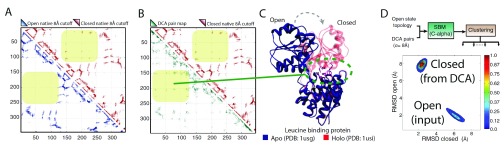
Direct coupling analysis (DCA) contact maps derived from protein family sequence co-evolution are consistent not only with single native structures but also with multiple functional configurations. ( **A**) Leucine-binding protein (LBP) contact maps derived from crystal structures: “open” without ligand and “closed” bound to a ligand. Each triangular region in the map shows a mark if residue pairs are less than 8 Å apart in the experimental structure. The closed contact map (upper triangle) has additional contacts not present in the open structure. ( **B**) The DCA contact map inferred from residue co-evolution (lower triangle) contains a superset of contacts from both open and closed conformations. ( **C**) A cartoon representation of the aligned open (apo) and closed (holo) LBP structures shows a large conformational change upon ligand binding. ( **D**) A structure-based model (SBM) is defined from the apo structure plus contact potentials stabilizing DCA contacts that are not already in the open structure. A two-dimensional root mean square deviation (RMSD) distribution of the states explored by molecular dynamics simulations of this hybrid Hamiltonian shows two peaks within 2 Å of the open and closed states. This shows the ability to uncover functional states via co-evolutionary couplings.

In addition to homo-multimerization, the set of conformations encoded in HDP contact maps can include functional motions. Multi-domain proteins can undergo conformational changes, for example, to accommodate ligands ^40^ or in response to phosphorylation ^41^. In periplasmic ligand binding proteins, there exists an open, ligand-free configuration and a closed, ligand-bound configuration. Molecular dynamics simulations can be performed by using an SBM specific to an open configuration but overlaid with an additional potential term consisting of a set of attractive, short-range interactions for each HDP ^27^. Figure 1 illustrates an example of this for the leucine-binding protein. The native contact maps for two crystal structures of leucine-binding protein are shown in Figure 1A: “open” without ligand and “closed” with ligand. The closed contact map has additional contacts not present in the open structure. The DCA contact map, shown as the lower triangular map in Figure 1B, contains a superset of both the open and closed configuration contacts. An SBM is constructed that is specific to the open structure ( Figure 1D) and additionally contains contact potentials stabilizing all the “non-native” DCA contacts (i.e., any DCA contacts that are not already in the open structure). These additional contacts are each given a stabilizing potential with a minimum at 8 Å. Molecular dynamics simulations of this hybrid SBM+DCA Hamiltonian show two clusters, each within 2 Å of either the open or closed state. Overlaying the DCA contacts does not disrupt the stability of the open structure, and additionally reveals the closed state without including any information from the closed crystal structure. This shows that co-evolutionary information can be used to uncover intermediary, hidden, and functionally relevant conformational states present in many protein families ^27^.

So far, we have discussed how HDP contact maps can be used for structural modeling. However, the fundamental output of the DCA algorithm is not direct information about co-evolving pairs but rather a Potts model Hamiltonian describing the effective energies of interaction for all pairs of residues in a protein family. This Hamiltonian, though not transferable to any sequences outside the family, should, in principle, be able to provide a quantitative window into the stabilities provided by each amino acid in a protein. Strong evidence of the utility of the effective energies comes from their ability to predict the stability changes of single-site mutants ^42, 43^ and significant correlations to folding rates ^44^. Including the so-called single-site fields in addition to the pairwise energies provides even better predictive power ^45^. These results suggested that the pairwise energies calculated from co-evolution could be used to inform thermodynamic models of protein folding. Indeed, folding simulations using SBMs with DCA-weighted native contact potentials can better capture transition state ensembles ^46^. DCA energies have also been shown to correlate with physical potentials when summed over the entire sequence ^47^. Confidence in the ability to estimate energies at both the single-mutant and full-sequence levels is allowing novel methods for investigating the effective energy landscape of evolution, and bridging the gap between biophysics and sequence evolution ^47, 48^. These developments are important for integrating the energetics of protein folding and function with protein evolution and selection, which will be crucial to understanding drug resistance and cancer development going forward.

## Future directions

The marriage between co-evolutionary information and physical models of biomolecules has been shown to be a fertile research field, where the most important results are yet to come. This field has been focused on rigorously validating the connection and usefulness between evolutionary information with structural modeling and experimental information. However, the true utility of co-evolutionary information is that it allows us to go places that are hard to access by current experimental technologies; important examples are those of membrane protein structure ^22, 23^ and dynamics, systems with transient conformational states, as well as investigation of large molecular assemblies that resist crystallographic characterization. Although crystal structures exist for FtsH AAA peptidase and the 30S ribosome, recent studies on these two systems ^28, 49^ show the promise of co-evolutionary information for discovering structural constraints in molecular assemblies. The ability to detect relevant evolutionary interactions has repercussions to our understanding of biomolecular assembly and function. Hopefully, these new tools can be used to alter protein conformation and rewire their interfaces. This has potential applications in the field of protein engineering, as well as systems biology. There is no conceptual hurdle to resisting the application of these ideas to RNA structure and function as well as protein-RNA interactions. Ultimately, we would hope to use all this knowledge to tackle biomedical problems that would help advance human health.

## Abbreviations

DCA, direct coupling analysis; DI, direct information; HDP, high direct information pair; MSA, multiple sequence alignment; SBM, structure-based model.

## References

[1] Bryngelson JD Wolynes PG: Spin glasses and the statistical mechanics of protein folding. *Proc Natl Acad Sci U S A.* 1987;84(21):7524–8. 10.1073/pnas.84.21.7524 3478708 PMC299331

[2] Leopold PE Montal M Onuchic JN: Protein folding funnels: a kinetic approach to the sequence-structure relationship. *Proc Natl Acad Sci USA.* 1992;89(18):8721–5. 10.1073/pnas.89.18.8721 1528885 PMC49992

[3] Onuchic JN Wolynes PG: Theory of protein folding. *Curr Opin Struct Biol.* 2004;14(1):70–5. 10.1016/j.sbi.2004.01.009 15102452

[4] Socci ND Onuchic JN Wolynes PG: Diffusive dynamics of the reaction coordinate for protein folding funnels. *J Chem Phys.* 1996;104(15):5860–8. 10.1063/1.471317

[5] Clementi C Nymeyer H Onuchic JN: Topological and energetic factors: what determines the structural details of the transition state ensemble and "en-route" intermediates for protein folding? An investigation for small globular proteins. *J Mol Biol.* 2000;298(5):937–53. 10.1006/jmbi.2000.3693 10801360

[6] Noel JK Whitford PC Sanbonmatsu KY: SMOG@ctbp: simplified deployment of structure-based models in GROMACS. *Nucleic Acids Res.* 2010;38(Web Server issue):W657–61. 10.1093/nar/gkq498 20525782 PMC2896113

[7] Morcos F Pagnani A Lunt B: Direct-coupling analysis of residue coevolution captures native contacts across many protein families. *Proc Natl Acad Sci U S A.* 2011;108(49):E1293–301. 10.1073/pnas.1111471108 22106262 PMC3241805

[8] Weigt M White RA Szurmant H: Identification of direct residue contacts in protein-protein interaction by message passing. *Proc Natl Acad Sci U S A.* 2009;106(1):67–72. 10.1073/pnas.0805923106 19116270 PMC2629192

[9] Taylor WR Sadowski MI: Structural constraints on the covariance matrix derived from multiple aligned protein sequences. *PLoS One.* 2011;6(12):e28265. 10.1371/journal.pone.0028265 22194819 PMC3237328

[10] Jones DT Buchan DW Cozzetto D: PSICOV: precise structural contact prediction using sparse inverse covariance estimation on large multiple sequence alignments. *Bioinformatics.* 2012;28(2):184–90. 10.1093/bioinformatics/btr638 22101153

[11] Kamisetty H Ovchinnikov S Baker D: Assessing the utility of coevolution-based residue-residue contact predictions in a sequence- and structure-rich era. *Proc Natl Acad Sci U S A.* 2013;110(39):15674–9. 10.1073/pnas.1314045110 24009338 PMC3785744

[12] Ekeberg M Lövkvist C Lan Y: Improved contact prediction in proteins: using pseudolikelihoods to infer Potts models. *Phys Rev E Stat Nonlin Soft Matter Phys.* 2013;87(1):012707. 10.1103/PhysRevE.87.012707 23410359

[13] de Juan D Pazos F Valencia A: Emerging methods in protein co-evolution. *Nat Rev Genet.* 2013;14(4):249–61. 10.1038/nrg3414 23458856

[14] Whitford PC Sanbonmatsu KY Onuchic JN: Biomolecular dynamics: order-disorder transitions and energy landscapes. *Rep Prog Phys.* 2012;75(7):076601. 10.1088/0034-4885/75/7/076601 22790780 PMC3695400

[15] Noel JK Onuchic JN: The Many Faces of Structure-Based Potentials: From Protein Folding Landscapes to Structural Characterization of Complex Biomolecules. In: Dokholyan NV, editor. *Computational Modeling of Biological Systems* Springer US;2012;31–54. 10.1007/978-1-4614-2146-7_2

[16] Hills RD Jr Brooks CL 3rd: Insights from coarse-grained Gō models for protein folding and dynamics. *Int J Mol Sci.* 2009;10(3):889–905. 10.3390/ijms10030889 19399227 PMC2672008

[17] Sułkowska JI Morcos F Weigt M: Genomics-aided structure prediction. *Proc Natl Acad Sci U S A.* 2012;109(26):10340–5. 10.1073/pnas.1207864109 22691493 PMC3387073

[18] Marks DS Colwell LJ Sheridan R: Protein 3D structure computed from evolutionary sequence variation. *PLoS One.* 2011;6(12):e28766. 10.1371/journal.pone.0028766 22163331 PMC3233603

[19] Marks DS Hopf TA Sander C: Protein structure prediction from sequence variation. *Nat Biotechnol.* 2012;30(11):1072–80. 10.1038/nbt.2419 23138306 PMC4319528

[20] Taylor WR Jones DT Sadowski MI: Protein topology from predicted residue contacts. *Protein Sci.* 2012;21(2):299–305. 10.1002/pro.2002 22102360 PMC3324774

[21] Nugent T Jones DT: Accurate *de novo* structure prediction of large transmembrane protein domains using fragment-assembly and correlated mutation analysis. *Proc Natl Acad Sci U S A.* 2012;109(24):E1540–7. 10.1073/pnas.1120036109 22645369 PMC3386101

[22] Hopf TA Colwell LJ Sheridan R: Three-dimensional structures of membrane proteins from genomic sequencing. *Cell.* 2012;149(7):1607–21. 10.1016/j.cell.2012.04.012 22579045 PMC3641781

[23] Wang Y Barth P: Evolutionary-guided *de novo* structure prediction of self-associated transmembrane helical proteins with near-atomic accuracy. *Nat Commun.* 2015;6: 7196. 10.1038/ncomms8196 25995083 PMC4833009

[24] Okazaki K Koga N Takada S: Multiple-basin energy landscapes for large-amplitude conformational motions of proteins: Structure-based molecular dynamics simulations. *Proc Natl Acad Sci U S A.* 2006;103(32):11844–9. 10.1073/pnas.0604375103 16877541 PMC1567665

[25] Whitford PC Miyashita O Levy Y: Conformational transitions of adenylate kinase: switching by cracking. *J Mol Biol.* 2007;366(5):1661–71. 10.1016/j.jmb.2006.11.085 17217965 PMC2561047

[26] Noel JK Schug A Verma A: Mirror images as naturally competing conformations in protein folding. *J Phys Chem B.* 2012;116(23):6880–8. 10.1021/jp212623d 22497217

[27] Morcos F Jana B Hwa T: Coevolutionary signals across protein lineages help capture multiple protein conformations. *Proc Natl Acad Sci U S A.* 2013;110(51):20533–8. 10.1073/pnas.1315625110 24297889 PMC3870752

[28] Jana B Morcos F Onuchic JN: From structure to function: the convergence of structure based models and co-evolutionary information. *Phys Chem Chem Phys.* 2014;16(14):6496–507. 10.1039/c3cp55275f 24603809

[29] Dago AE Schug A Procaccini A: Structural basis of histidine kinase autophosphorylation deduced by integrating genomics, molecular dynamics, and mutagenesis. *Proc Natl Acad Sci U S A.* 2012;109(26):E1733–42. 10.1073/pnas.1201301109 22670053 PMC3387055

[30] Schug A Weigt M Onuchic JN: High-resolution protein complexes from integrating genomic information with molecular simulation. *Proc Natl Acad Sci U S A.* 2009;106(52):22124–9. 10.1073/pnas.0912100106 20018738 PMC2799721

[31] Casino P Rubio V Marina A: Structural insight into partner specificity and phosphoryl transfer in two-component signal transduction. *Cell.* 2009;139(2):325–36. 10.1016/j.cell.2009.08.032 19800110

[32] Tamir S Rotem-Bamberger S Katz C: Integrated strategy reveals the protein interface between cancer targets Bcl-2 and NAF-1. *Proc Natl Acad Sci U S A.* 2014;111(14):5177–82. 10.1073/pnas.1403770111 24706857 PMC3986192

[33] Zheng W Schafer NP Davtyan A: Predictive energy landscapes for protein-protein association. *Proc Natl Acad Sci U S A.* 2012;109(47):19244–9. 10.1073/pnas.1216215109 23129648 PMC3511104

[34] dos Santos RN Morcos F Jana B: Dimeric interactions and complex formation using direct coevolutionary couplings. *Sci Rep.* 2015;5: 13652. 10.1038/srep13652 26338201 PMC4559900

[35] Ovchinnikov S Kamisetty H Baker D: Robust and accurate prediction of residue-residue interactions across protein interfaces using evolutionary information. *eLife.* 2014;3:e02030. 10.7554/eLife.02030 24842992 PMC4034769

[36] Hopf TA Schärfe CP Rodrigues JP: Sequence co-evolution gives 3D contacts and structures of protein complexes. *eLife.* 2014;3:e03430. 10.7554/eLife.03430 25255213 PMC4360534

[37] Liu Y Eisenberg D: 3D domain swapping: as domains continue to swap. *Protein Sci.* 2002;11(6):1285–99. 10.1110/ps.0201402 12021428 PMC2373619

[38] Yang S Cho SS Levy Y: Domain swapping is a consequence of minimal frustration. *Proc Natl Acad Sci U S A.* 2004;101(38):13786–91. 10.1073/pnas.0403724101 15361578 PMC518834

[39] Brown JH: Breaking symmetry in protein dimers: designs and functions. *Protein Sci.* 2006;15(1):1–13. 10.1110/ps.051658406 16373473 PMC2242361

[40] Felder CB Graul RC Lee AY: The Venus flytrap of periplasmic binding proteins: an ancient protein module present in multiple drug receptors. *AAPS PharmSci.* 1999;1(2):E2. 10.1208/ps010202 11741199 PMC2761117

[41] Lätzer J Shen T Wolynes PG: Conformational switching upon phosphorylation: a predictive framework based on energy landscape principles. *Biochemistry.* 2008;47(7):2110–22. 10.1021/bi701350v 18198897

[42] Lui S Tiana G: The network of stabilizing contacts in proteins studied by coevolutionary data. *J Chem Phys.* 2013;139(15):155103. 10.1063/1.4826096 24160546

[43] Cheng RR Morcos F Levine H: Toward rationally redesigning bacterial two-component signaling systems using coevolutionary information. *Proc Natl Acad Sci U S A.* 2014;111(5):E563–71. 10.1073/pnas.1323734111 24449878 PMC3918776

[44] Mallik S Kundu S: Co-evolutionary constraints of globular proteins correlate with their folding rates. *FEBS Lett.* 2015;589(17):2179–85. 10.1016/j.febslet.2015.06.032 26162935

[45] Contini A Tiana G: A many-body term improves the accuracy of effective potentials based on protein coevolutionary data. *J Chem Phys.* 2015;143(2):25103. 10.1063/1.4926665 26178131

[46] Cheng RR Raghunathan M Noel JK: Constructing sequence-dependent protein models using coevolutionary information. *Protein Sci.* 2016;25(1):111–22. 10.1002/pro.2758 26223372 PMC4815312

[47] Morcos F Schafer NP Cheng RR: Coevolutionary information, protein folding landscapes, and the thermodynamics of natural selection. *Proc Natl Acad Sci U S A.* 2014;111(34):12408–13. 10.1073/pnas.1413575111 25114242 PMC4151759

[48] Sikosek T Chan HS: Biophysics of protein evolution and evolutionary protein biophysics. *J R Soc Interface.* 2014;11(100):20140419. 10.1098/rsif.2014.0419 25165599 PMC4191086

[49] Mallik S Akashi H Kundu S: Assembly constraints drive co-evolution among ribosomal constituents. *Nucleic Acids Res.* 2015;43(11):5352–63. 10.1093/nar/gkv448 25956649 PMC4477670

